# Moving Mountains—The BRCA1 Promotion of DNA Resection

**DOI:** 10.3389/fmolb.2019.00079

**Published:** 2019-09-03

**Authors:** Ruth M. Densham, Joanna R. Morris

**Affiliations:** Birmingham Centre for Genome Biology, Institute of Cancer and Genomic Sciences, Medical and Dental Schools, University of Birmingham, Birmingham, United Kingdom

**Keywords:** BRCA1, SMARCAD1, USP48, 53BP1, resection, homologous recombination

## Abstract

DNA double-strand breaks (DSBs) occur in our cells in the context of chromatin. This type of lesion is toxic, entirely preventing genome continuity and causing cell death or terminal arrest. Several repair mechanisms can act on DNA surrounding a DSB, only some of which carry a low risk of mutation, so that which repair process is utilized is critical to the stability of genetic material of cells. A key component of repair outcome is the degree of DNA resection directed to either side of the break site. This in turn determines the subsequent forms of repair in which DNA homology plays a part. Here we will focus on chromatin and chromatin-bound complexes which constitute the “mountains” that block resection, with a particular focus on how the breast and ovarian cancer predisposition protein-1 (BRCA1) contributes to repair outcomes through overcoming these blocks.

## Introduction

DNA DSBs occur as a result of exogenous agents such as irradiation and chemotherapy, but also as a result of cellular processes, such as replication and transcription. A single-ended DSB may be formed when replication forks encounter single-stranded DNA (ssDNA) breaks. In contrast, some cell types generate DSBs as part of specialist processes such as immunoglobulin gene rearrangements and recombination in meiosis. Experimentally, DSBs are also induced by rare-cutting restriction enzymes, such as the *I-Sce1* endonuclease, and sequence-guided nucleases, such as clusters of regularly interspaced short palindromic repeats (CRISPR) and CRISPR associated protein 9 (CRISPR-Cas9).

**Figure 1:**
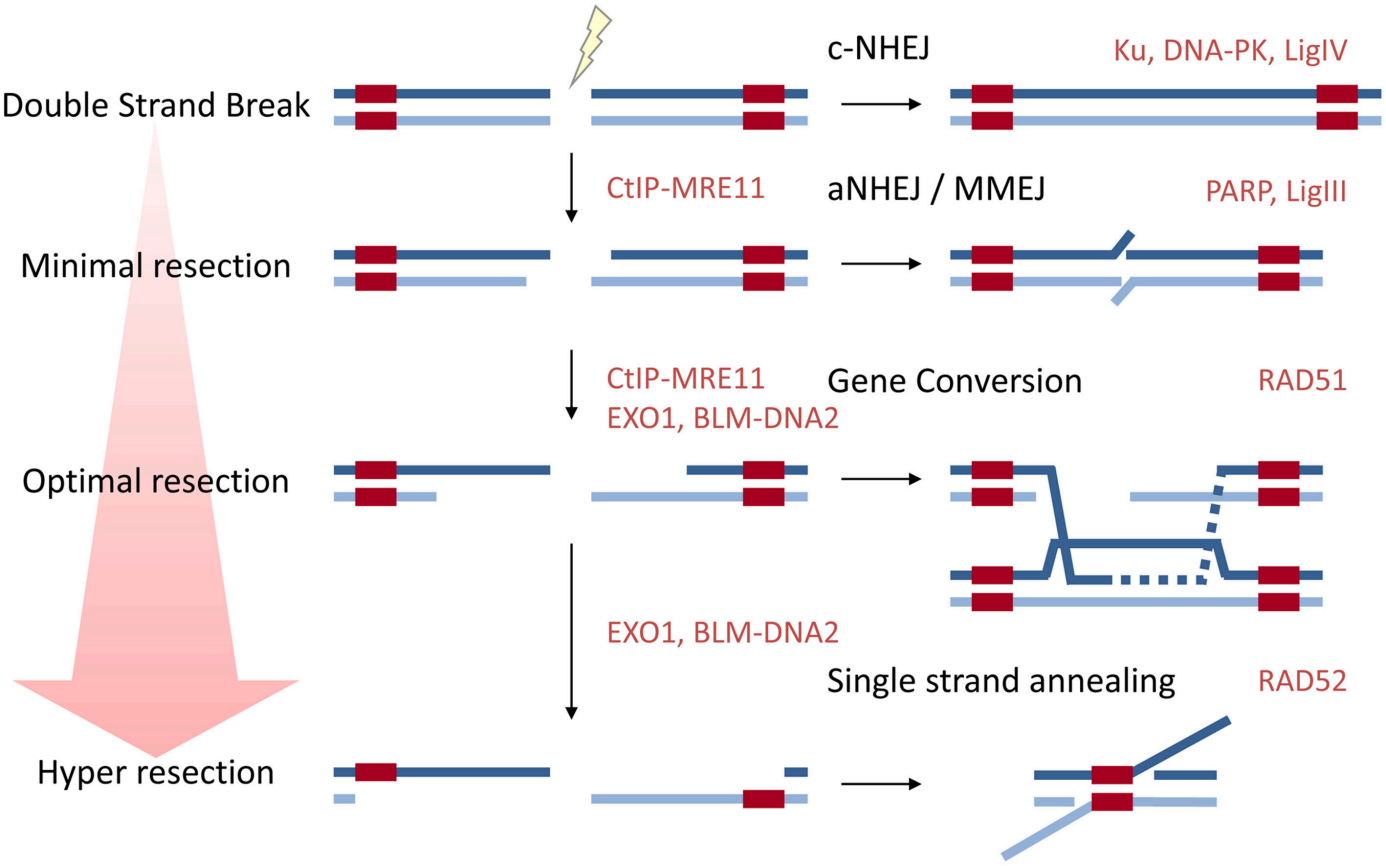
A diagram to show how resection influences repair pathway choice. Approximately 80% of DSBs are repaired by classical NHEJ which does not require resection. aNHEJ or MMEJ requires minimal resection to expose regions of microhomology. Long range resection is required for the major HDR pathways gene conversion (GC) and single-strand annealing (SSA). Key proteins in each pathway are given in red.

DSBs in mammalian cells can be repaired by several means (Figure 1). Non-homologous end joining (NHEJ) involves the re-ligation of both ends of the break, and may or may not involve nucleic processing of the ends and polymerases to fill gaps prior to ligation to restore the backbone. NHEJ is rapid, predominates throughout the cell cycle, and undertakes the majority (~80%) of DSBs repair (Mao et al., 2008; Beucher et al., 2009). When no end-processing occurs and the two correct ends are ligated this is error-free, but if the ends are processed or incorrect ends are ligated, material is lost or translocations occur. Alternative non-homologous end joining (aNHEJ), or microhomology-mediated end joining (MMEJ) is used when one of the core-components of NHEJ are absent (such as Ku70/80 or Ligase IV), aNHEJ uses short patches of microhomology (<25 nucleotides) so that minimal resection of either end is required (Seol et al., 2018). Homology-directed repair (HDR) requires a template to copy from and all HDR pathways share the same initial step of resection around the DNA DSB to create long 3′ ssDNA overhangs coated by the ssDNA binding protein replication protein A (RPA). When resection exposes direct repeats either side of the break, i.e., homologous sequences, repair can occur following direct annealing of the repeat sequences, in a process catalyzed by the DNA Repair Protein RAD52 Homolog (RAD52). This process of single-strand annealing (SSA) is error prone due to deletion of the intervening sequence between the direct repeats (reviewed in Bhargava et al., 2016). In the form of HDR often referred to as “homologous recombination,” herein called gene conversion (GC), the ssDNA participates in a homology search followed by DNA strand invasion. The critical step is the formation of the ssDNA-RAD51 [DNA Repair Protein RAD51 Homolog (RecA Homolog, *E. Coli*)] nucleofilament in which RPA loaded onto ssDNA exposed by resection either side of the DSB, is exchanged for RAD51. This ssDNA-RAD51 nucleofilament invades the homologous sister chromatid, displacing one strand of DNA and forming a synapse with the homologous sequence on the other strand in a DNA-loop (D-loop). The invading strand then acts as a primer for polymerases to extend along the template. Depending on how the structure is resolved determines whether the chromatids gain material from the partner or not: if the D-loop is dissolved they do not; but if the two crossed-over structures (Holliday junctions) are cleaved, cross-over products are formed in half of cases. GC is often referred to as “error free” as it uses the sister chromatid as the template and no genetic material is lost. Heterologous recombination, i.e., the use of near-homologous sequences is suppressed by Regulator of telomere elongation helicase 1 (RTEL1) and Bloom Syndrome RecQ-like helicase (BLM) (Leon-Ortiz et al., 2018).

The process of digesting one strand on the duplex on either side of the DSB, resection, is initiated by the endonuclease activity of Meiotic recombination 11 homolog A (MRE11)—a DSB repair nuclease, found in a complex with Nijmegen breakage syndrome 1/Nibrin (NBS1) and the DSB repair protein RAD50 Homolog (RAD50) [termed MRN (MRE11-RAD50-NBS1) complex] together with the carboxy-terminal binding protein interacting protein (CtIP) (Sartori et al., 2007; Stracker and Petrini, 2011; Anand et al., 2016; Daley et al., 2017). CtIP forms a tetramer and appears to have a preference for binding blocked DNA ends (Wilkinson et al., 2019), which may provide some explanation for the preferential use of HDR in tackling lesions resulting from Poly-ADP-Ribose-Polymerase (PARP) inhibition or topoisomerase-poisons, which produce DNA-protein adducts. The endonuclease cut of MRE11 occurs around 20–40 nucleotides from the blocked end (Anand et al., 2016, 2019). Intriguingly MRN-CtIP is found constitutively in an inactive form in complex with the resection repressor Cell cycle and apoptosis regulator 2 (CCAR2). The CtIP-CCAR2 interaction is disrupted locally on damaged chromatin and also by phosphorylation of CtIP (Lopez-Saavedra et al., 2016). Indeed the CtIP-MRN complex is subject to several post-translational modifications. Phosphorylation by cyclin dependent kinases in S/G2 promotes CtIP-MRN association and MRE11 activity (Huertas and Jackson, 2009; Buis et al., 2012; Orthwein et al., 2015; Anand et al., 2016), thereby limiting resection to when the sister chromatid template is present. After MRE11 endonuclease activity forms a DNA nick, the 3′-5′ exonuclease activity of MRE11 degrades back to the break site (Shibata et al., 2014; Cejka, 2015). This short-range resection is also promoted by another recently described nuclease, Exonuclease 3′-5′ domain containing 2 (EXD2) (Broderick et al., 2016; Nieminuszczy et al., 2016). CtIP is critical for resection and its depletion is sufficient to switch some HDR-committed breaks to repair by NHEJ (Shibata et al., 2011, 2014) indicating that, at least in some cases, lesions that might have undergone HDR can be re-directed to NHEJ. Long-range resection is performed 5′-3′ by two redundant pathways: the dominant pathway through Exonuclease-1 (Exo1); and a backup pathway of BLM and DNA replication helicase/nuclease 2 (DNA2) (Gravel et al., 2008; Mimitou and Symington, 2008; Zhu et al., 2008; Nimonkar et al., 2011; Tomimatsu et al., 2012; Myler et al., 2016). Exo1 generates extensive 3′ ssDNA (Cejka, 2015; Myler et al., 2016), while the RecQ-helicase BLM (and to a lesser degree the Werner Syndrome RecQ Like Helicase) unwinds dsDNA, and the nuclease DNA2 is a ssDNA flap endonuclease without specificity to one end or the other (Kim et al., 2006). The ssDNA produced is bound by RPA which protects the 3′ end from DNA2 attack, since DNA2 can only displace RPA from the 5′-end to enable degradation (Niu et al., 2010; Nimonkar et al., 2011; Zhou et al., 2015).

In cells DNA breaks rarely occur on naked DNA, but on nucleic acid wrapped in chromatin and chromatin and chromatin signaling has emerged as a key aspect controlling the vital step of DNA resection that in turn determines the downstream repair strategy employed.

## Chromatin Barriers to Resection

DNA is wound round an octamer of histones that make up the nucleosome. The basic nucleosome consists of two copies of each of the core histones Histone 2A (H2A), Histone 2B (H2B), Histone 3 (H3), and Histone 4 (H4). The linker histone, Histone 1 (H1), binds at the DNA entry and exit point, stabilizes nucleosomes, and can thereby promote higher-order chromatin architecture. There are multiple variants for each histone providing a complex array of variations in nucleosome structure that occurs at the level of histone composition. In addition, all histones have long flexible N-terminal tails that extend away from the nucleosome body and which are highly modified by post-translational modifications (reviewed in Armeev et al., 2018). Chromatin context is crucial for DNA repair outcome and the first challenge the cellular machinery meets is to deal with the underlying chromatin structure. Nucleosomes can block the progression of Exo1 *in vitro* (Adkins et al., 2013) and in yeast the heterogeneity of resection lengths has been at least partly attributed to the disruption of Exo1-resection by the position of nucleosomes (Mimitou et al., 2017).

It is perhaps surprising then that in mammalian cells immediately following DNA damage chromatin undergoes a rapid, but transient compaction in the environment local to the DSB. This has been visualized recently using Fluorescence lifetime imaging microscopy (FLIM)- Förster resonance energy transfer (FRET) techniques with fluorescently labeled H2B to assess nuclear-wide chromatin compaction and compaction around laser-induced DSBs. Local chromatin compaction around the break site is observed within 10 min of damage (Lou et al., 2019). This initial repressive state may prevent unwanted movement of the DSB keeping a relationship between DNA ends, act to strip the chromatin of irrelevant factors and prime the modification landscape ready for new alterations. It is clear that it is required for local transcriptional silencing (reviewed in Gursoy-Yuzugullu et al., 2016). A number of mechanisms drive this transient compaction including PARP-dependent recruitment of heterochromatin protein 1 (HP1), KRAB-associated protein-1 (KAP1), macroH2A variants, and methyltransferases (reviewed in Price and D'Andrea, 2013; Oberdoerffer, 2015). This initial repressive state must necessarily be overcome in order to permit repair. In FLIM-FRET analysis the kinase ataxia-telangiectasia mutated (ATM) and E3 ligase RING finger-8 (RNF8) regulate chromatin de-compaction and compact chromatin at later time points is found beyond the boundary of the repair locus (Lou et al., 2019).

Tri-methylated-lysine 9 Modified H3 domains (H3K9me3) generated adjacent to the break, bind and activate the lysine(K) acetyltransferase 5 (KAT5 also known as TIP60) (Sun et al., 2009; Ayrapetov et al., 2014). In turn, KAT5 acetylates and activates the master regulator of the DNA damage response, ATM (Sun et al., 2009) and also modifies the H4 tail. Acetylation of the H4 tail blocks the interaction of the tail with the acidic groove on adjacent nucleosome patches, facilitating a more open chromatin structure (reviewed in Price and D'Andrea, 2013). Once activated ATM disrupts the small ubiquitin like modifier (SUMO)-mediated interaction of KAP1 with Chromodomain helicase DNA binding protein 3 (CHD3), a member of the histone deacetylase complex referred to as the Nucleosome Remodeling Deacetylase (NuRD) complex (Goodarzi et al., 2011). KAP1 depletion can relieve chromatin compaction and allow subsequent repair (Ziv et al., 2006), and similarly the ATM-mediated CHD3 dissociation from chromatin, promotes chromatin relaxation and allows DNA repair. In addition, both the SUMO targeting E3 ubiquitin (ub) ligase RING Finger 4 (RNF4) and Valosin-containing protein/AAA+-type ATPase p97 (VCP/p97) interact with pS824-KAP1-SUMO. VCP/p97 can extract ubiquitinated proteins from membranes or cellular structures, or segregate them from binding proteins and RNF4-VCP/p97 promotes removal and degradation of SUMOylated KAP1 (Kuo et al., 2014), providing a further mechanism for chromatin de-compaction.

The canonical modification catalyzed by the RING Finger 20/RING Finger 40 dimer (RNF20-RNF40), H2B-K120ub, is associated with transcription in open chromatin (Nickel and Davie, 1989; Zhu et al., 2005). This modification is also induced by RNF20-RNF40 following DNA damage (Moyal et al., 2011; Nakamura et al., 2011), where the H2B-K120ub mark is required for recruitment of subsequent DNA repair factors, such as BRCA1 and RAD51 (Moyal et al., 2011; Nakamura et al., 2011). The requirement is likely to be an indirect effect of H2B-K120ub in promoting chromatin relaxation. Indeed, relaxation relieves the requirement for RNF20 in HDR (Nakamura et al., 2011). H2B-K120ub supports increased access to DNA by promoting both local and higher order chromatin de-compaction (Fierz et al., 2011; Debelouchina et al., 2017).

A further means to relieve histone-repression of resection is in the recruitment of chromatin remodelers to break sites. The INO80 chromatin remodeler complex promotes incorporation of the histone variant H2AZ, which in turn promotes an open chromatin structure, in part through facilitating H4 acetylation (Xu et al., 2012). Similarly the yeast “remodels the structure of chromatin,” RSC, complex contributes to MRX and Ku recruitment to damage sites (reviewed in Chambers and Downs, 2012). In humans the SWI/SNF-related, matrix associated, actin-dependent regulator of chromatin, subfamily A, member 4 (SMARCA4 also known as BRG1), which is the ATPase subunit of the SWI/SNF-B polybromo-associated BRG1-associated factor (PBAF) chromatin remodeling complex, is required for RPA-RAD51 exchange (Qi et al., 2015), while the component, AT-Rich Interaction Domain 2 (ARID2), promotes RAD51 recruitment through direct protein interaction (de Castro et al., 2017). The SWI/SNF-related, matrix associated, actin-dependent regulator of chromatin, subfamily A, member 5 (SMARCA5 also called SNF2H) which is the catalytic subunit of ISWI chromatin remodeling complexes recruits to DNA damage sites through PARP1 and Sirtuin 6 (SIRT6) activity and through the structural Nuclear mitotic apparatus protein (NuMA) (Smeenk et al., 2013; Toiber et al., 2013; Vidi et al., 2014). The co-factor of SMARCA5, Remodeling and spacing factor-1 (RSF-1), similarly recruits to sites of damage, and does so in an ATM-dependent fashion (Min et al., 2014). In turn SMARCA5-dependent remodeling, for example of heterochromatin, requires H2B-ubiquitination by RNF20/RNF40 (Klement et al., 2014). Intriguingly, at least some of these remodelers share an interaction domain for binding nucleosomes in order to induce nucleosome sliding. For example INO80 and SMARCA5 require the acidic patch of H2A/B (Gamarra et al., 2018) that is also an interaction face for the unacetylated H4 tail, and for other signaling and repair factors, suggesting a mutually exclusive and perhaps sequential hierarchy of remodeling events directing repair responses.

The differences between repair in open, active euchromatin compared to closed, repressive heterochromatin have been reviewed elsewhere (Murray et al., 2012; Watts, 2016). For many years the view that a more open chromatin environment of euchromatin is conducive to HDR and that breaks within transcribed genes are repaired more frequently by HDR has persisted (Aymard et al., 2014; Lemaitre et al., 2014). Indeed a recent study using CRISPR-Cas9 to target specific loci found that open-chromatin may recruit insufficient p53 binding protein 1 (53BP1) (van den Berg et al., 2019), required to promote NHEJ and restrict HDR. Additionally, a more nuanced view has recently arisen in which repair choice is actively directed in different chromatin environments. For example, the chromatin-binding protein Lens epithelium-derived growth factor (LEDGF) binds preferentially to epigenetic methyl-lysine histone markers characteristic of active transcription and also interacts with CtIP in a damage dependent way, thereby improving resection within active genomic regions (Daugaard et al., 2012). In addition, specialist remodelers, such as the Snf2-like remodeler Helicase, lymphoid specific (HELLS), appear to enable HDR at some heterochromatic regions (Kollarovic et al., 2019) and heterochromatin-resident proteins such as HP1 and Sentrin/SUMO-Specific Protease SENP7 (SENP7) nevertheless facilitate HDR (Garvin et al., 2013; Lee et al., 2013). A recent study using CRISPR-Cas9 to quantify HDR- and NHEJ-derived gene editing events at single-target sequences subjected to distinct chromatin conformations found that NHEJ and not HDR was more sensitive to chromatin state. Reduction of Cas9 activity in G1 was a far greater determinant of the relationship between NHEJ and HDR than chromatin conformation (Janssen et al., 2019).

### Chromatin Signaling as a Barrier to Resection

When both strands of DNA are broken nearby, sheering the chromosome, a dramatic signaling cascade occurs to initiate repair. This cascade, often referred to as the DNA damage response (DDR), is reviewed extensively elsewhere (Jackson and Bartek, 2009; Altmeyer and Lukas, 2013; Setiaputra and Durocher, 2019) while signaling leading to 53BP1-Shieldin recruitment to damage sites is described briefly here. DSBs are detected by two protein complexes, the Ku70/80 dimer and the MRN-complex. MRN (MRE11-RAD50-NBS1) tethers to the two ends and recruits the serine/threonine kinase ATM through interaction with NBS1. Phosphorylation of the histone variant H2AX at serine-139, recruits the Mediator of DNA damage checkpoint 1 (MDC1), which in turn recruits more ATM, amplifying the signal either side of the dsDNA break. The E3 ub ligase RNF8 is recruited to damage sites by interaction with ATM-phosphorylated-MDC1. Once at DSBs, RNF8 modifies the linker histone H1 with K63-ub chains (Thorslund et al., 2015). Additionally L3MBTL Histone methyl-lysine binding protein 2 (L3MBTL2) may be recruited by MDC1 and also modified by RNF8 (Nowsheen et al., 2018). H1 modification is assisted by the HECT, UBA, and WWE domain containing E3 ub ligase 1 (HUWE1) (Mandemaker et al., 2017), and the polycomb repressor complex 1 (PRC1) (Ismail et al., 2013). RNF8 signaling promotes the recruitment of another E3 ub ligase, RING finger 168 (RNF168), which binds K63-ub chains (Doil et al., 2009; Stewart et al., 2009; Panier et al., 2012), and also interacts with the nucleosome acidic patch where it catalyzes mono-ubiquitination of H2A at K13/K15 to promote recruitment of 53BP1 (Doil et al., 2009; Mattiroli et al., 2012; Fradet-Turcotte et al., 2013). H2A N-terminal ubiquitination is critical for 53BP1 accumulation, where the H2AK15-conjugated ub acts to trap a portion of 53BP1 against the nucleosome surface (Wilson et al., 2016). KAT5 acetylation of H4K16 reduces the second mode of 53BP1 interaction with nucleosomes, interaction of its Tudor domain with H4K20me2 (Tang et al., 2013), while acetylation at H2AK15 blocks 53BP1 binding as this modification is mutually exclusive with H2AK15ub (Jacquet et al., 2016). Binding of Bromo-domain containing 2 (BRD2) to acetylated H4 protects the chromatin from histone deacetylases (HDACs) 2 Kb both sides of the break, and limits the 53BP1 competitive inhibitor L3MBTL Histone methyl-lysine binding protein 1 (L3MBTL1) from binding (Dhar et al., 2017; Gursoy-Yuzugullu et al., 2017). RNF168 recognizes its own H2AK13/K15ub mark and thereby auto-propagates this signal along chromatin (Chen J. et al., 2012; Panier et al., 2012; Poulsen et al., 2012).

**Figure 2:**
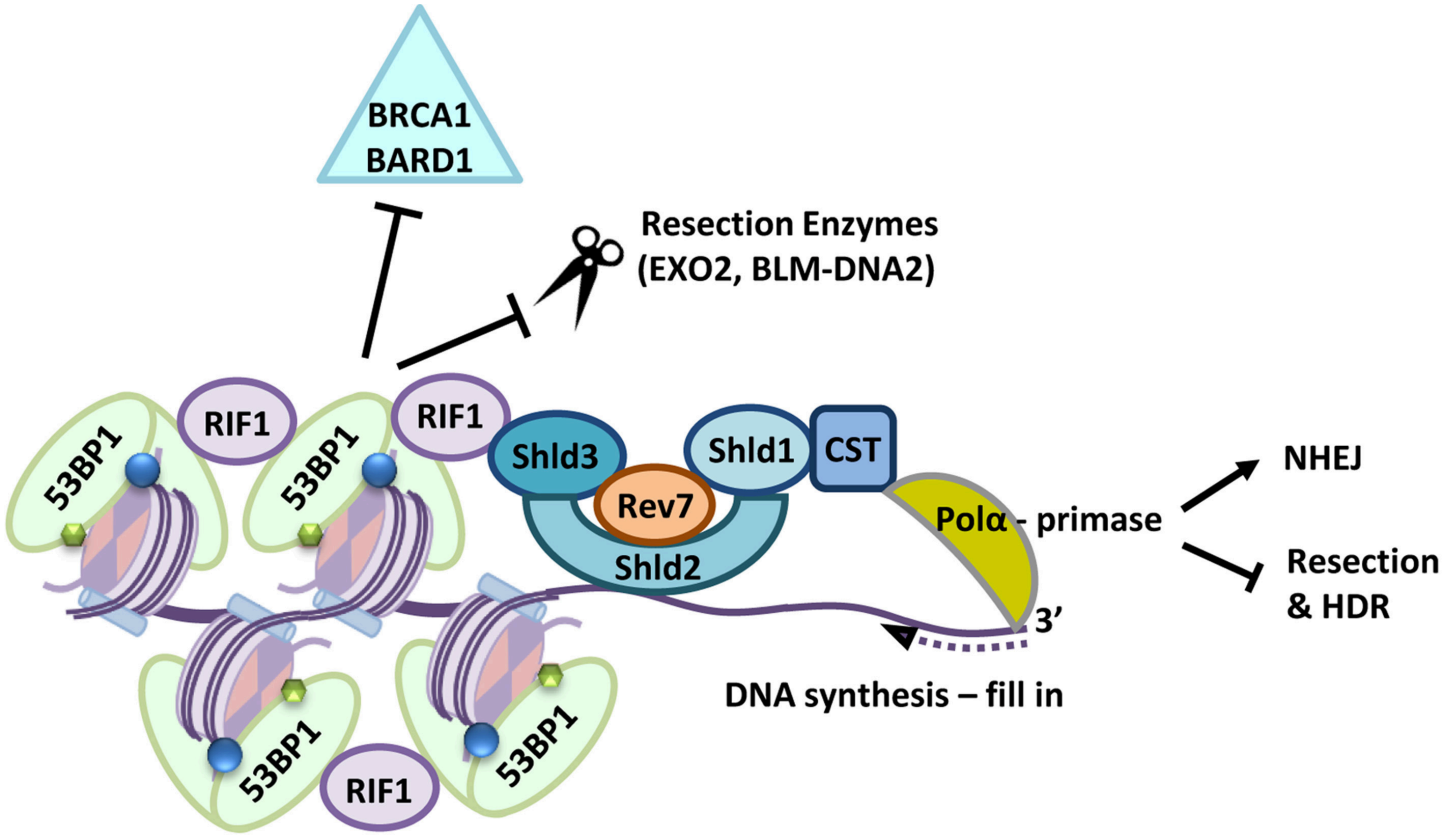
Following a DSB 53BP1 interacts with modified histones, (H2A-K15-ubiquitin blue circles, H4K20-dimethylation, green hexagons), the 53BP1-RIF1-Shieldin (Shld1-Shld2-Shld3-Rev7-CST) complex is recruited to sites of DNA damage where it also prevents retention of BRCA1-BARD1. Shld2 binds directly to ssDNA stretches >50 nucleotides long via three OB-folds. Together the Shieldin complex recruits DNA Polα which in turn primes DNA synthesis to fill in resected DNA ends. This prevents long range resection and repair by HDR pathways and supports repair by NHEJ.

53BP1 is heavily phosphorylated by ATM following damage and the phosphorylated protein interacts with two apparently independent effectors. PAX transcription activation domain interacting protein-1-like (PTIP) which in turn interacts with Artemis (Munoz et al., 2007; Wang et al., 2014) and RIF1 Replication timing regulatory factor 1 (RIF1) which interacts with the Shieldin complex (Manke et al., 2003; Silverman et al., 2004; Munoz et al., 2007; Wu et al., 2009; Chapman et al., 2013; Daley and Sung, 2013; Di Virgilio et al., 2013; Escribano-Diaz et al., 2013; Feng et al., 2013; Zimmermann et al., 2013; Wang et al., 2014; Boersma et al., 2015; Tomida et al., 2015, 2018; Xu et al., 2015; Bakr et al., 2016; Bluteau et al., 2016; Dev et al., 2018; Ghezraoui et al., 2018; Gupta et al., 2018; Mirman et al., 2018; Noordermeer et al., 2018; Zlotorynski, 2018). The identification of Shieldin has been an exciting advance in understanding how chromatin signaling acts to inhibit resection (reviewed in Setiaputra and Durocher, 2019). RIF1 bound to 53BP1 interacts with Shld3/RINN1-Rev7 and in turn Shld2/RINN2-Shld1/RINN3 (Names: Shieldin Complex Subunit 3/RINN1-REV7-Interacting Novel NHEJ Regulator 1, REV7 Homolog/ Mitotic Arrest Deficient 2 Like 2; Shieldin Complex Subunit 1/RINN3-REV7-Interacting Novel NHEJ Regulator 3; Shieldin Complex Subunit 2/RINN2-REV7-Interacting Novel NHEJ Regulator 2; and Shieldin Complex Subunit 1/RINN3-REV7-Interacting Novel NHEJ Regulator 3, respectively). Shld2 carries 3 OB folds which interact with ssDNA and are required for the promotion of 53BP1 mediated NHEJ and HDR inhibition. Surprisingly Shld2 can precipitate ssDNA of >50 nucleotides, but not smaller than 30 nucleotides (Dev et al., 2018; Findlay et al., 2018; Gao et al., 2018; Noordermeer et al., 2018), which is slightly greater than the length of minimally resected DNA. Shieldin in turn contacts a complex made up of CST telomere replication complex component 1 (CTC1)-Subunit of CST Complex (STN1)-Telomere Length Regulation Protein TEN1 Homolog (TEN1), known as the CST complex, together with DNA polymerase-alpha (Pol-α). CST-Pol-α is critical to the integrity of telomeres, where it performs C-strand fill in (reviewed in Stewart J. A. et al., 2018) and CST-pol-α appears to perform a similar role at resected DNA ends, filling in the short regions of resected DNA (Barazas et al., 2018; Mirman et al., 2018) so that 53BP1 and its effectors may not only block resection but also reverse it. This mechanism provides further options for repair; since filling in the region that has been processed by MRE11 potentially generates a “clean” end for NHEJ (Setiaputra and Durocher, 2019; illustrated in Figure 2). Indeed 53BP1 is required for the promotion of several forms of NHEJ, including class-type switching, a subset of VDJ recombination and the fusion of unprotected telomere ends (Manis et al., 2004; Ward et al., 2004; Nakamura et al., 2006; Difilippantonio et al., 2008; Dimitrova et al., 2008; Kibe et al., 2016). Dramatically, the impact of the 53BP1-complex on suppressing resection is clearest in cells lacking BRCA1 (Bothmer et al., 2010, 2011; Bouwman et al., 2010; Bunting et al., 2010).

The embryonic lethality of *Brca1* deficient mice is rescued by concurrent loss of *53bp1* and coincides with improved DNA resection, and improved measures of HDR-proficiency, such as RAD51 foci in irradiated cells, PARP inhibitor resistance and improved repair of integrated HDR-substrates together with a reduction of radial chromosomes, often referred to as a hallmark of toxic end joining (Cao et al., 2009; Bouwman et al., 2010; Bunting et al., 2010, 2012; Li et al., 2016; Nacson et al., 2018). These data demonstrate that BRCA1, of itself, is not needed for the promotion of resection, since the need for the protein is largely overcome by loss of 53BP1, but show BRCA1 is critical to overcoming the resection block mediated by 53BP1. A similar, though less potent, relationship is also clear between BRCA1 and members of the 53BP1-Shieldin complex (Chapman et al., 2013; Di Virgilio et al., 2013; Escribano-Diaz et al., 2013; Feng et al., 2013; Zimmermann et al., 2013; Boersma et al., 2015; Tomida et al., 2015, 2018; Xu et al., 2015; Dev et al., 2018; Findlay et al., 2018; Ghezraoui et al., 2018; Gupta et al., 2018; Mirman et al., 2018; Noordermeer et al., 2018; Zlotorynski, 2018).

### Repair Structures

In G1-phase cells the 53BP1 protein is found as a dense focus around the DSB, whereas in S-phase cells 53BP1 accumulations are less dense and more dispersed from the focus center. At the central focus core of damage sites in S-phase cells BRCA1, CtIP and the ssDNA binding protein RPA are found (Chapman et al., 2012; Kakarougkas et al., 2013). These S-phase structures are large, with 53BP1 peak density mapping in an axis through a foci center as far as 1 μm across, presumably ~0.5 μm from the DSB (Chapman et al., 2012; Kakarougkas et al., 2013). Loss of BRCA1 reduces the circumference of the 53BP1 localization, placing it in the center of the foci, resembling a G1-phase focus (Chapman et al., 2012; Kakarougkas et al., 2013). Thus BRCA1 plays a role in the physical localization of 53BP1, contributing to its placement away from the break core in S-phase cells.

**Figure 3:**
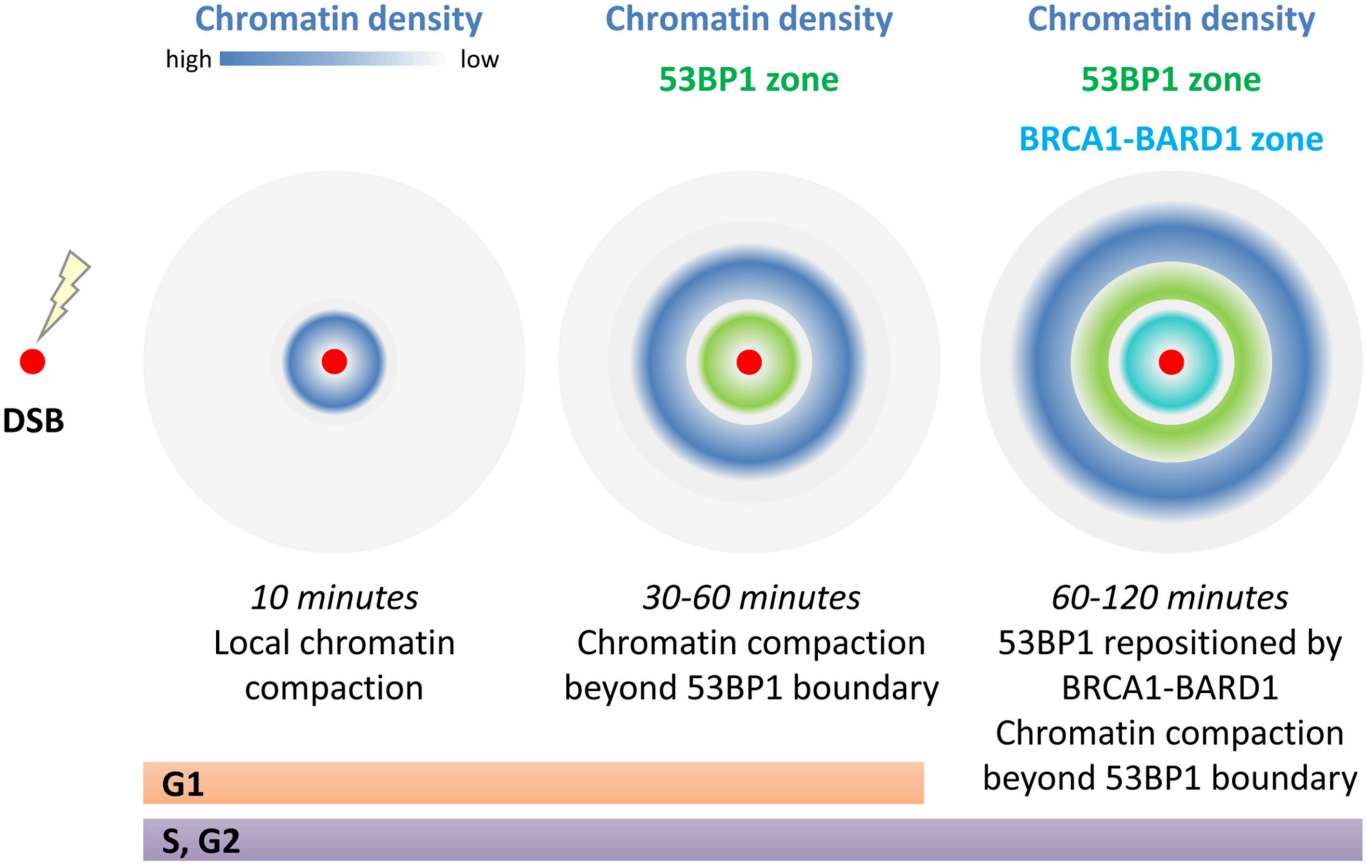
Chromatin compaction around a single DSB changes with time. Immediately following damage (within 10 min) local compaction occurs which has been linked to transcriptional repression, limiting movement of the break ends, and to strip and prime chromatin modifications for repair. At 30–60 min post repair chromatin density is at its greatest beyond the 53BP1 boundary that marks the break site. In S/G2, the 53BP1 boundary is repositioned by BRCA1-BARD1 to open up the damage site for long range resection. In addition the 53BP1 damage complexes are thought to have liquid like phase properties which may be key to these large scale (5 μm diameter) effects on chromatin densities.

Mapping of chromatin compaction reveals that substantial local chromatin changes accompany the repair response. The chromatin density is re-arranged so that the initial compaction seen proximal to the break at 10 min post damage is lost and at 30–60 min a ring of condensed chromatin forms further away from the break site, with the greatest density occurring beyond the regions bound by 53BP1. Chromatin peak density mapping through focus centers reveal that compaction occurs as much as 5 μm apart, presumably ~2.5 μm from the DSB (Lou et al., 2019; illustrated in Figure 3). These large chromatin rearrangements are dependent on ATM and RNF8 (Lou et al., 2019). One speculative explanation for the chromatin “wave” beyond 53BP1 is that chromatin remodeling required to promote long-range resection, forces chromatin bunching outside the resected region (see below). Another possibility is that the liquid-like properties of 53BP1 assemblies, which show fusion and sensitivity to disruption of hydrophobic interactions by detergents (Kilic et al., 2019), displace chromatin. These later observations are particularly fascinating in view of reports that liquid phase-separation mechanically excludes chromatin as it grows (Shin et al., 2018). Understanding the role of remodeling factors and the three dimensional chromatin structures of both the damaged and template strands within the repair structures is needed to address what these structures represent and the reason for their large scale.

### BRCA1 Regulation of Resection

BRCA1 exists as an obligate heterodimer with its N-terminal binding partner BRCA1-associated RING domain protein 1 (BARD1) and in the absence of BARD1, BRCA1 is degraded (Joukov et al., 2006). The BRCA1-BARD1 heterodimer has the ability to act as an E3 ub ligase by improving the transfer of ub from an interacting and loaded E2 ub conjugating enzyme to target lysines (Brzovic et al., 2003). Several E2 conjugating enzymes interact with the BRCA1-RING domain, and not the BARD1-RING domain, to catalyze the generation of ub conjugates (Christensen et al., 2007). Nevertheless, BARD1 brings more than protein stability to BRCA1, contributing a charged residue that interacts with ub to facilitate its transfer from the loaded E2 (Densham et al., 2016). Several targets of the BRCA1-BARD1 E3 ligase activity have been identified (reviewed in Wu et al., 2008) including several independent reports of H2A modification (Mallery et al., 2002; Zhu et al., 2011; Kalb et al., 2014a). Residues at the extreme C-terminus of H2A at K125/K127/K129 have been mapped as those modified (Kalb et al., 2014a). Modeling and mutagenesis approaches have suggested that BRCA1-BARD1 contacts the H2A/B nucleosome acidic patch via an arginine anchor to promote ubiquitination of H2A (Buchwald et al., 2006; McGinty et al., 2014).

Ubiquitin modification of H2A at K118/K119 is associated with transcriptional repression (Blackledge et al., 2014; Kalb et al., 2014b), and de-repression of satellite DNA has been reported in human and mouse BRCA1-deficient cancers (Zhu et al., 2011). Recently cancer-associated germline patient variants in the BARD1-RING have been described which do not reduce BRCA1-BARD1 ligase activity, but do specifically prevent ubiquitination of H2A (Stewart M. D. et al., 2018). These mutations also suppress transcriptional repression, resulting in activation of estrogen metabolism genes in MCF10A breast cells (Stewart M. D. et al., 2018). Whether the de-repression of transcription can impact HDR directly is not clear, but there is potential for re-expressed genes, such as those involved in estrogen metabolism or satellite RNA to increase the demand for HDR (Santen et al., 2015; Kishikawa et al., 2018) the latter through the generation of RNA: DNA hybrids at repeat sequences and at replication forks (Zhu et al., 2018; Padeken et al., 2019).

Cells lacking BRCA1 are sensitive to a broad range of DNA damaging agents (reviewed in Costes and Lambert, 2012; Zimmermann and de Lange, 2013; Ceccaldi et al., 2016). However in a human cell system complemented with ligase defective BRCA1-BARD1, cells were sensitive to the PARP inhibitor, Olaparib, and the Topoisomerase inhibitor, camptothecin, but not sensitive to replication stressing agents, hydroxyurea, or aphidicolin (Densham et al., 2016). BRCA1 E3 ligase defective chicken DT40 cells also are sensitive to Topoisomerase inhibitors (Sato et al., 2012) and neither these cells, nor similarly altered mouse cells, nor human cells complemented with ligase defective BRCA1-BARD1, show sensitivity to DNA interstrand cross-linking agents (Reid et al., 2008; Sato et al., 2012; Densham et al., 2016) (although the engineered mouse cells do exhibit increased chromosome aberrations after cross-linking agent treatment Reid et al., 2008). BRCA1 loss, or loss of the ligase function, is associated with reduced long-range resection (Shibata et al., 2011, 2014; Alagoz et al., 2015; Densham et al., 2016; Drost et al., 2016). Intriguingly E3 ligase proficiency also correlates with the ability to position 53BP1 away from the break site in S-phase cells (Densham et al., 2016). Thus a subset of BRCA1-mediated responses relate to resection and to 53BP1 positioning.

**Figure 4:**
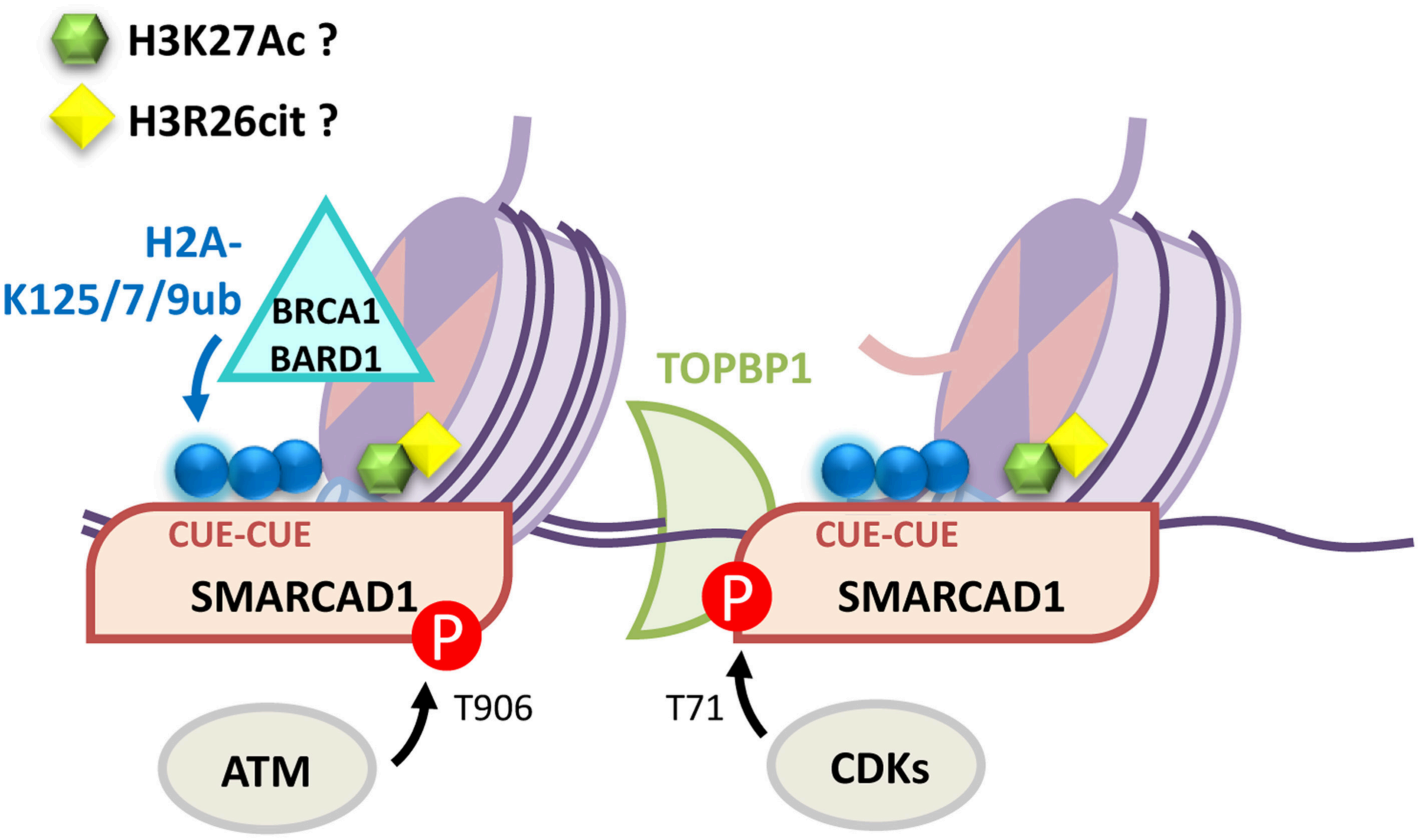
Multiple mechanisms contribute to SMARCAD1 recruitment to DNA damage sites. BRCA1-BARD1 modification of H2A-K125/127/129ub is recognized by SMARCAD1 CUE domains. Phosphorylation events by ATM (SMARCAD1-T906) facilitate recruitment and CDKs (SMARCAD1-T71) promote TOPBP1 interaction. SMARCAD1 preferentially binds and is activated by ssDNA-nucleosomes. Finally, SMARCAD1 has been proposed to directly bind H3K27Ac and H3R26cit although the role of these interactions in the DDR has yet to be characterized.

Amongst the remodelers critical to DNA repair in yeast is the SNF2 family ATPase SWI/SNF-related, matrix-associated actin-dependent regulator of chromatin, subfamily A, containing DEAD/H Box-1 (SMARCAD1) homolog, Fun30. Fun30 promotes long range resection at camptothecin-induced lesions by facilitating the activity of Exonuclease 1 (Exo1) (Chen X. et al., 2012; Costelloe et al., 2012; Eapen et al., 2012). Significantly this remodeler is less important for resection in the absence of histone-bound Rad9, the 53BP1 ortholog, which like 53BP1 acts to block 5′ strand processing (Chen X. et al., 2012; Adkins et al., 2013). SMARCAD1 has two N-terminal ub-binding CUE domains (coupling of ub to ER degradation) (Kang et al., 2003; Shih et al., 2003) and these link BRCA1-BARD1 ligase function and H2A modification to 53BP1 positioning and resection (Densham et al., 2016). Moreover SMARCAD1 ATPase activity and the integrity of its ub binding domains are required for HDR repair and for the positioning of 53BP1 away from the BRCA1 core (Densham et al., 2016). These observations point to ub driven SMARCAD1 remodeling, rather than 53BP1:BRCA1 competition at chromatin, as critical to 53BP1 positioning. CUE domain interactions with ub are typically weak, with reported dissociation constants ranging from 20 to 160 μM (Kang et al., 2003; Prag et al., 2003; Shih et al., 2003) and while SMARCAD1 CUE domains and BRCA1-BARD1 are required for full SMARCAD1 recruitment to damage sites (Densham et al., 2016) an ATM consensus site at SMARCAD1-T906 is also required (Chakraborty et al., 2018). In addition, a recent peptide array screen has shown that SMARCAD1 binds to histone 3 modifications, including citrullinated Histone 3. Citrullination occurs when an arginine is deaminated and converted to the amino acid citrulline. SMARCAD1 binds modified Histone 3: H3R26Cit> H3K27ac>H3R17Cit>H3R26me2 (Xiao et al., 2017). Intriguingly H2A-K127/K129ub and H3R26Cit/K27ac are proximal on the nucleosome surface presenting the possibility that SMARCAD1 interaction with histone is through combined post-translational modification interactions. In yeast the CDK-mediated phosphorylation of Fun30 promotes interaction with Dpb11 [homolog of DNA Topoisomerase II Binding Protein 1 (TOPBP1)] and Mec1-Ddc2 [orthologs of ataxia telangiectasia and Rad3-related protein (ATR) and ATR-interacting protein (ATRIP)] resulting in improved Fun30 recruitment to damaged chromatin in S-phase. In human cells TOPBP1 similarly interacts through phosphorylated SMARCAD1 (Bantele et al., 2017). Further, purified Fun30 binds nucleosomes wrapped in ssDNA preferentially over dsDNA-wrapped nucleosomes and ssDNA-nucleosomes are effective at activating Fun30 (Adkins et al., 2017), providing a potential means for short-range resection to activate the remodeler. Taken as a whole, recent evidence suggest a model in which several components of SMARCAD1 recruitment prime it to locating and activating, not only at DNA break sites, but at minimally resected DNA (illustrated in Figure 4).

Intriguingly, independently of ubiquitination, SMARCAD1 constitutively interacts with KAP1 directly through its first CUE domain (Rowbotham et al., 2011; Ding et al., 2018; Lim et al., 2019). SMARCAD1 co-purifies with several other remodeling factors (Rowbotham et al., 2011) associated with gene silencing and heterochromatin formation, some of which have also been implicated in 53BP1 repositioning (Alagoz et al., 2015).

How SMARCAD1/Fun30 in turn promotes remodeling of 53BP1/Rad9 is less clear. Fun30 can promote nucleosome sliding or eviction of H2A-H2B from nucleosomes (Awad et al., 2010). Sliding might be expected to contribute to the compaction wave of condensed chromatin observed outside of 53BP1 domains (Lou et al., 2019) but this is not mutually exclusive with the model of 53BP1 phase separation and chromatin exclusion (Shin et al., 2018; Kilic et al., 2019). The related remodeler SMARCA5/SNF2h shifts DNA discontinuously with movement on the entry side preceding its exit (Sabantsev et al., 2019). A recent Cryo-electron microscopy model of a SMARCA5 dimer on nucleosomes suggests the disordered H2A-H2B acidic patch inhibits the second SMARCA5 protomer, while disorder near the bound SMARCA5 dyad stimulates directional DNA translocation (Armache et al., 2019). Thus, we might speculate that order induced by protein-protein interaction at the nucleosome acidic patch, for example by the bound 53BP1-ubiquitylation-dependent recruitment motif, could influence remodeling directionality or proficiency.

In the context of the DNA damage response the BRCA1-BARD1 E3 ub ligase contributes the third ub modification of H2A. Indeed H2A modification in a nucleosomal context is remarkably site specific both for the E3 ligases responsible and for the readers of these marks (reviewed in Uckelmann and Sixma, 2017). H2A has long tails at both N- and C-termini which can be modified by conjugation of ub at three major sites: K13/K15 by RNF168 (Mattiroli et al., 2012), K118/K119 by the PRC1 (Nickel and Davie, 1989), and K125/K127/K129 by BRCA1-BARD1 (Kalb et al., 2014a). The majority of H2A ubiquitination in the cell is at the K119 site (Nickel and Davie, 1989) which is ubiquitinated by proteins that form part of the PRC1 and the mark is associated with transcriptional gene repression and heterochromatin (Wang et al., 2004). Many de-ubiquitinating enzymes (DUBs) have been implicated in the removal of ub from H2A (reviewed in Vissers et al., 2008; Uckelmann and Sixma, 2017) but none had previously been reported to be specific for the BRCA1-H2Aub mark.

*In vitro* work from the group of Prof. Titia Sixma identified the highly conserved Ubiquitin Specific Peptidase 48 (USP48) (human has 77% identify with Xenopus, 95% with mouse Usp48) as a DUB specific for nucleosomal-H2A substrates and, more specifically, for nucleosomal-H2A modified at the BRCA1 K125/K127/K129 sites (Uckelmann et al., 2018). Additionally, like Ubiquitin Specific Peptidase 14 (USP14), the proteasome-associated DUB, USP48 requires a second “auxiliary” ub (i.e., not the substrate ubiquitin) to achieve full catalytic potential. This “auxiliary” ub can be at either the H2A-K125/K127/K129ub or H2A-K118/K119ub sites but it only increases activity toward ub removal from the three BRCA1 targeted sites, i.e., USP48 does not cleave H2A-K118/K119ub when H2A-K125/K127/K129ub is present (Uckelmann et al., 2018).

**Figure 5:**
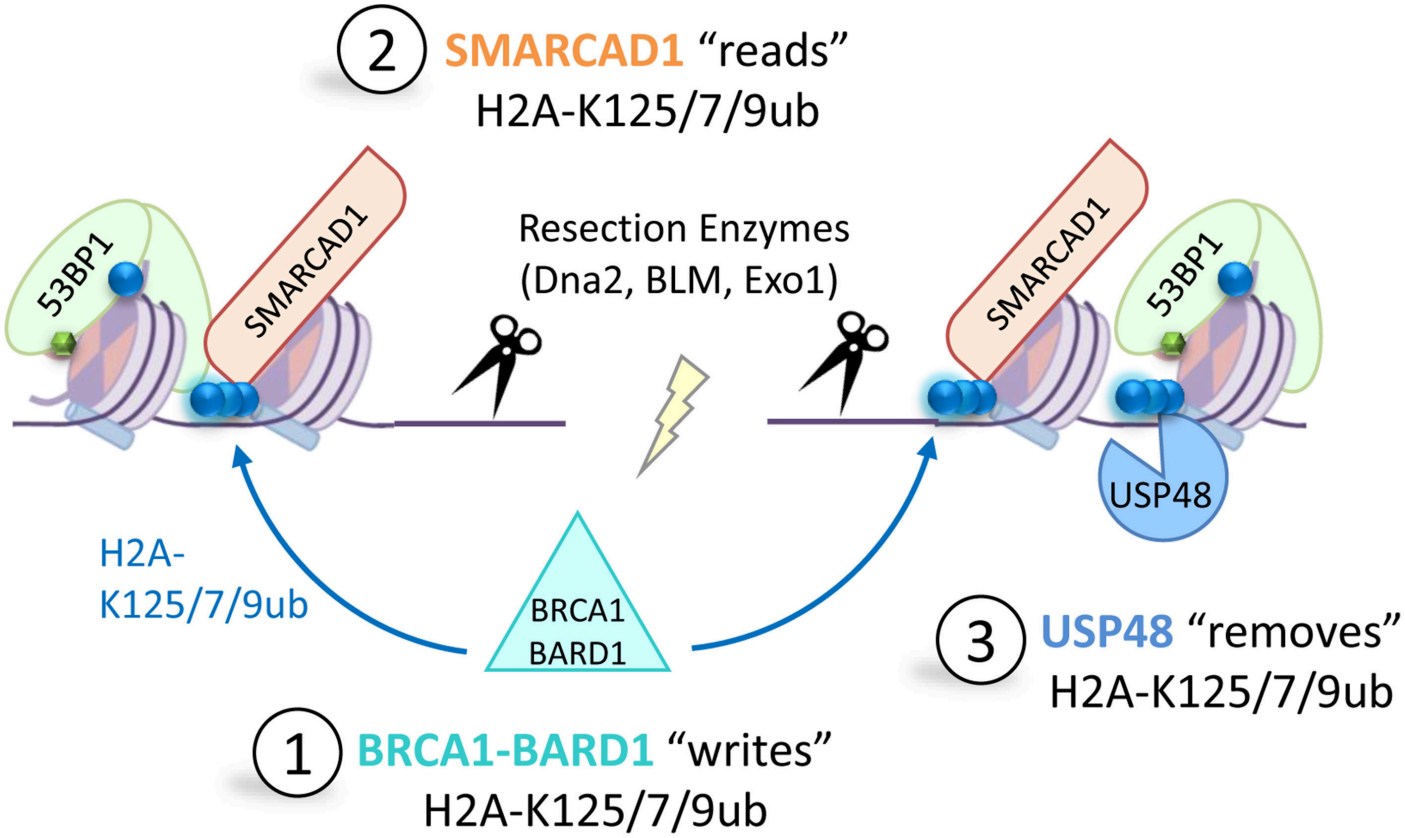
A new BRCA1-circuit that controls DNA repair pathway choice. In S-phase BRCA1-BARD1 is retained at DNA double strand break sites where it mono-ubiquitinates the extreme C-terminus of H2A at K125/127/129 (1). This ubiquitination modification is recognized by the chromatin remodeler SMARCAD1 (2) which remodels nucleosomes to promote 53BP1 repositioning at the break site (53BP1 binds modified H2A-K15-ubiquitin blue circles, H4K20-dimethylation, green hexagons). This allows recruitment of long-range resection enzymes, such as DNA2, BLM or EXO1, required for homology-directed repair. The deubiquitinating enzyme USP48 specifically removes the BRCA1-mediated H2A-Ub modification (3) to prevent over-resection and limit use of the mutagenic single-strand annealing repair pathway.

Modulating the levels of USP48 dramatically influences DNA resection lengths. Over-expression of USP48 results in restricted resection whereas low USP48 levels result in placement of 53BP1 further from the damage site and result in the extension of BRCA1 and SMARCAD1 dependent resection. The removal or depletion of 53BP1 results in extended resection lengths to the degree that SSA is favored over GC, leading to the suggestion that 53BP1 acts as to limit the extent of resection (Ochs et al., 2016). Intriguingly cells depleted of USP48 develop a dependence on SSA DNA repair even though they have normal levels of 53BP1 (Uckelmann et al., 2018). These findings suggest cells may fine-tune 53BP1 placement and HDR mechanisms through the opposing activities of the BRCA1-BARD1 ligase and USP48 DUB (illustrated in Figure 5). Additionally cells lacking the Fanconi anemia compatibility component A (FANCA), show improved survival to interstrand cross linking agents when they lack USP48 (Velimezi et al., 2018). These cells have enhanced BRCA1 dependent clearance of DNA damage that appears unrelated to resection proficiency (Velimezi et al., 2018). We speculate that BRCA1-BARD1 ligase function, amplified by loss of USP48, may provide a back-up role for the Fanconi anemia core complex.

The degree to which the BRCA1-BARD1-USP48 relationship is significant in regulating resection-driven repair pathway choice will be dependent on how the pathway is modulated in different environments. The identity of the “auxiliary” ub site on H2A is not clear and the ligase responsible for the modification not known. Similarly it is unclear if SMARCAD1: chromatin interaction favors ub-modification at any of one of the three lysines of H2A-K125/K127/K129 over another. It is possible that the dependency of USP48 for an auxiliary ub has the potential to regulate the degree of resection in particular chromatin environments; for example, in regions of heterochromatin marked by PRC1 mediated modification at H2A-K118/K119.

Given the potential for mutagenic DNA repair conferred by hyper-resection many regulatory mechanisms are to be expected. For example, incorporation of H2AZ at sites of damage has been proposed to limit resection and define chromatin boundaries (Xu et al., 2012). Interestingly, H2AZ, like H2AX, has shorter C-terminal tails than H2A and lacks the C-terminal lysines K125/K127/K129 present on H2A. H2AZ may thus be refractory to BRCA1 modification and SMARCAD1 remodeling.

In addition, positioning of 53BP1 by BRCA1 ligase activity is not the only means by which the block on resection is resisted. BRCA1 can counteract RIF1 recruitment in S-phase under conditions where no impact on 53BP1 is obvious (Chapman et al., 2013; Escribano-Diaz et al., 2013; Feng et al., 2013; Zimmermann et al., 2013). BRCA1 contributes to the recruitment of the protein phosphatase 4C (PP4C) to dephosphorylate 53BP1 and release RIF1 (Feng et al., 2015; Isono et al., 2017). BRCA1 is also reported to contribute to the recruitment of a further E3 ub ligase Ub-like with PHD and RING finger domains 1 (UHRF1), which mediates K63-linked polyubiquitination of RIF1, and results in its dissociation from 53BP1, thereby facilitating resection (Zhang et al., 2016). Further in S-phase cells RIF1 is gradually competed out from 53BP1 by the protein Suppressor of Cancer cell Invasion (SCAI), which binds 53BP1 to allow BRCA1-mediated repair (Isobe et al., 2017). In addition ATM and CDK2 control the chromatin remodeling activity of the SWI2-SNF2 remodeler, Cockayne syndrome group B (CSB), which interacts with RIF1 and remodels chromatin by evicting histones, which limits RIF1-REV7 but promotes BRCA1 accumulation (Batenburg et al., 2017).

## Concluding Remarks

The response to DNA breaks drives both dramatic and subtle local chromatin changes. That resection is sensitive to chromatin state has been utilized by cells to regulate resection lengths in and of itself and chromatin has been used as a substrate to build inhibitory blocks, or mountains, upon. BRCA1-BARD1 and TOPBP1 are part of a signaling milieu that places and initiates the chromatin remodeling activity of SMARCAD1 at the right place to reposition 53BP1, while several mechanisms counter the interaction of 53BP1 with RIF1. The degree of resection is the net result of nuclease-digestion vs. Shieldin-CST-Pol-α fill in, where the positioning of the fill-in machinery further from the break site appears to give nucleases the upper-hand. Unrestrained BRCA1-mediated remodeling can lead to hyper-resection and bias HDR mechanisms from accurate GC to mutagenic SSA.

We understand comparatively little about the relative physical positioning of many of the factors critical to the regulation of resection including the 53BP1-binding proteins responsible for the block on resection, those that promote resection, and only recently has relationship of chromatin with these factors begun to emerge. The explosion in the number of components capable of promoting and, in particular, restricting resection, illustrates the premium that the cell places on tuning appropriate resection lengths. Given the critical role it plays in repair pathway choice, how these factors are regulated will be key to understanding how chromatin context and HDR repair are interwoven.

## Author Contributions

All authors listed have made a substantial, direct and intellectual contribution to the work, and approved it for publication.

### Conflict of Interest Statement

The authors declare that the research was conducted in the absence of any commercial or financial relationships that could be construed as a potential conflict of interest.
